# Histone deacetylase 6 controls cardiac fibrosis and remodelling through the modulation of TGF‐β1/Smad2/3 signalling in post‐infarction mice

**DOI:** 10.1111/jcmm.70063

**Published:** 2024-09-04

**Authors:** Junqiao Fang, Shangzhi Shu, Hui Dong, Xueling Yue, Jinshun Piao, Shuyan Li, Lan Hong, Xian Wu Cheng

**Affiliations:** ^1^ Department of Cardiology and Hypertension, Jilin Provincial Key Laboratory of Stress and Cardiovascular Disease Yanbian University Hospital Yanji Jilin China; ^2^ Department of Cardiology, The Wuxi Fifth People's Hospital The Fifth Affiliated Hospital of Jiangnan University Wuxi Jiangshu China; ^3^ Department of Cardiology The First Hospital of Jilin University Changchun Jilin China; ^4^ Department of Physiology and Pathophysiology, College of Medicine Yanbian University Yanjin Jilin China; ^5^ Key Laboratory of Natural Medicines of the Changbai Mountain, Ministry of Education Yanbian University Yanji Jilin China

**Keywords:** cardiac fibroblast, cardiac fibrosis, collagen, histone deacetylases 6, myocardial infarction

## Abstract

Histone deacetylase 6 (HDAC6) belongs to the class IIb group of the histone deacetylase family, which participates in remodelling of various tissues. Herein, we sought to examine the potential regulation of HDAC6 in cardiac remodelling post‐infarction. Experimental myocardial infarction (MI) was created in HDAC6‐deficient (HDAC6^−/−^) mice and wild‐type (HADC6^+/+^) by left coronary artery ligation. At days 0 and 14 post‐MI, we evaluated cardiac function, morphology and molecular endpoints of repair and remodelling. At day 14 after surgery, the ischemic myocardium had increased levels of HADC6 gene and protein of post‐MI mice compared to the non‐ischemic myocardium of control mice. As compared with HDAC6^−/−^‐MI mice, HADC6 deletion markedly improved infarct size and cardiac fibrosis as well as impaired left ventricular ejection fraction and left ventricular fraction shortening. At the molecular levels, HDAC6^−/−^ resulted in a significant reduction in the levels of the transforming growth factor‐beta 1 (TGF‐β1), phosphor‐Smad‐2/3, collagen I and collagen III proteins and/or in the ischemic cardiac tissues. All of these beneficial effects were reproduced by a pharmacological inhibition of HADC6 in vivo. In vitro, hypoxic stress increased the expressions of HADC6 and collagen I and III gene; these alterations were significantly prevented by the HADC6 silencing and TubA loading. These findings indicated that HADC6 deficiency resists ischemic injury by a reduction of TGF‐β1/Smad2/3 signalling activation, leading to decreased extracellular matrix production, which reduces cardiac fibrosis and dysfunction, providing a potential molecular target in the treatment of patients with MI.

## 1 INTRIDUCTION

Cardiovascular disease (CVD) remains a significant cause of premature mortality and rising healthcare costs.^1^, ^2^ In 2019, CVD was the underlying cause of 9.6 million deaths among men and 8.9 million deaths among women, approximately one‐third of all deaths worldwide.^3^ It is well known that the adult mammalian heart, particularly the human heart, cannot regenerate after a myocardial infarction (MI), and a post‐MI fibrotic scar tissue develops to replace the dead cells. The subsequent remodelling of the surrounding myocardium leads to impaired cardiac function. The remodelling process includes thickening (hypertrophy) and stiffening (fibrosis) of the left ventricular (LV) wall.^4^ Post‐MI myocardial repair and remodelling are the result of a series of elaborate and complex activities, initiated by an intense aseptic inflammation and immune cell infiltration that serves to break down both the extracellular matrix (ECM) and clearly damaged or dead cells, followed by a reparative phase with the resolution of inflammation, myofibroblast proliferation, scar formation and neovascularization.^5^, ^6^ Few treatments have been proven to be effective against post‐MI fibrosis.

Histone acetylation is usually modulated by histone deacetylases (HDACs) and histone acetyltransferases, and this acetylation participates in the regulation of various gene expressions. HDACs are a large family of enzymes comprising four classes (I, IIa/b, III and IV); a total of 18 human HDACs have been identified according to functional and phylogenetic criteria, with HDAC6 belonging to the class IIb group.^7^, ^8^ Aberrant forms of multiple HDACs with altered acetylation levels are frequently observed in numerous human cancers.^9^ Over the last decade, there has been a wealth of studies investigated the roles of various HDAC isoforms in the development of CVD. Gene deletion and overexpression studies have revealed important functions of these enzymes in the pathological processes of cardiac remodelling, which involve hypertrophy, apoptosis, necrosis, metabolism, contractility and fibrosis.^10^ HDAC inhibitors have shown promise as therapeutic agents for CVD, reducing cardiac injury and pathological remodelling in preclinical models of MI.^11^, ^12^ Lemon and colleagues investigated whether HDAC6 could become a mediator of hypertension‐induced cardiac remodelling by applying selective HDAC inhibitors in multiple rodent models of hypertension.^13^ It has also been reported that a deletion of HDAC6 could prevent the development of cardiac dysfunction in both hypertrophic and fibrotic conditions.^14^ Transforming growth factor‐beta (TGF‐β1) is a vital regulator that participates in the development of fibrosis in many organs,^15^ and this has prompted us to further explore the relationship between HDAC6 and the TGF‐β pathway.

In this study, we used wild‐type (HDAC6^+/+^) mice, HDAC6 knockout (HDAC6^−/−^) mice and Tubastatin A (TubA, known as a potent and highly selective HDAC6 inhibitor^16^) to investigate the role(s) of HDAC6 in post‐MI cardiac remodelling and dysfunction, focusing on the TGF‐β1/Smad2/3 signalling pathway. In an in vitro experiment, we applied HDAC6 silencing and pharmacological inhibition to further explore the close interaction between HDAC6 and TGF‐β1/Smad2/3 signal in cultured rat neonatal cardiofibroblasts.

## 2 MATERIALS AND METHODS

### 2.1 Experimental animals

The animal experiments were reviewed and approved by the Institutional Animal Care and Use Committee at Yanbian University (Protocol: YD20211128016). Eight‐week‐old HDAC6^+/+^ male C57BL/6J (provided by Yanbian University Animal Center) and HDAC6^−/−^ mice provided by Shanghai Biomodel Organism Science & Technology Development Co. (Shanghai, China; protocol: 2022‐W5‐1109) that weighed 22–26 g were housed in an SPF‐level facility under a 12‐h light/dark cycle at 22 ± 3°C and a relative humid environment. All mice had free access to abundant food and water. The mice were monitored throughout the experimental period, and all experimental protocols involving the mice were performed by trained research staff.

### 2.2 MI model

The mouse MI model was created as described.^17^ In brief, HDAC6^+/+^ and HDAC6^−/−^ mice as the MI group were each anaesthetised with 5% isoflurane inhalation with an air delivery system. After the mice became unconscious, the anaesthetic agent was switched to 2%–3% isoflurane to maintain the anaesthesia status. A small incision was made over the left chest, and the fourth intercostal space was exposed. With a clamp slightly open, a small hole was formed and the heart was manually and smoothly squeezed out of the thoracic cavity. Next, 7–0 silk suture was used to ligated the left main descending coronary artery (LAD) of the mice of both genotypes (named the HDAC6^+/+^‐MI mice and HDAC6^−/−^‐MI mice) at the same level at the lower edge of the left atrium from the LAD's origin. The heart was then immediately returned back into the thoracic cavity. Sham‐operated mice of both genotypes underwent the same procedure without LAD ligation (named the HDAC6^+/+^‐sham and HDAC6^−/−^‐sham mice).

Each mouse in the two MI groups was given a daily peritoneal injection of Tub A, 10 mg/kg body weight (Apexbio Technology, Houston, TX, USA) or vehicle, from 12 h before the surgery until 7 days post‐MI.

### 2.3 Sample collections

**FIGURE 1:**
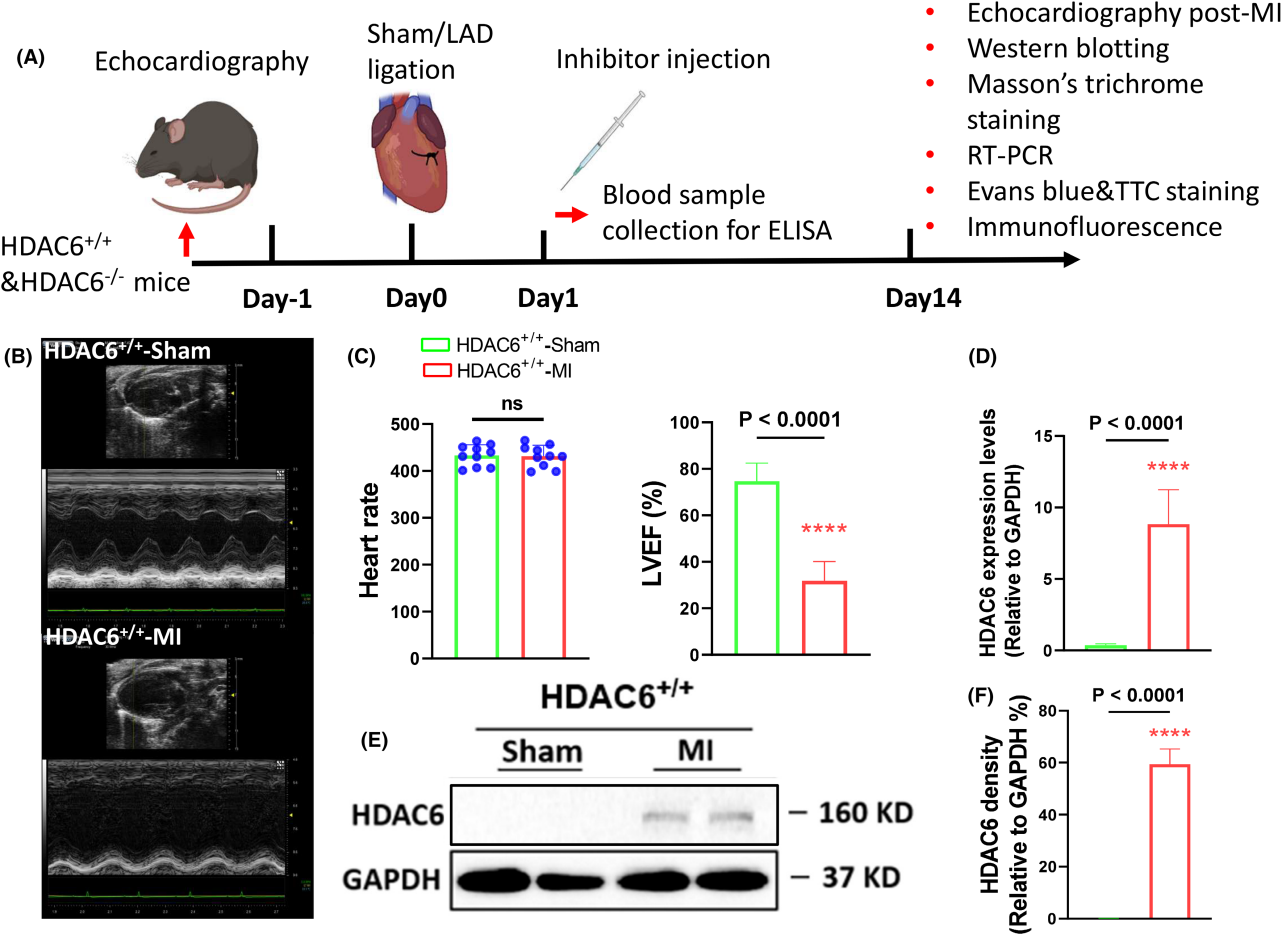
Ischemia increased the expression of histone deacetylase 6 (HDAC6) in the left ventricle (LV) tissues of HDAC6^+/+^‐MI mice. (A) Schematic illustration of the mouse myocardial infarction (MI) surgery and the treatment and sampling procedures at the indicated time points. Eight‐week‐old male HDAC6^+/+^ mice underwent a sham operation (HDAC6^+/+^‐Sham) or MI surgery (HDAC6^+/+^‐MI). (B, C) Heart rate (HR) and representative long‐axis LV M‐mode echocardiographic images and quantitative data showing the levels of the LV ejection fraction (LVEF), LV end‐diastolic diameter (LVDd) and LV end‐systolic diameter (LVSd) of both groups (*n* = 6 per group). (D, E) Representative Western blot images and quantitative data showing the levels of HADC6 proteins in the infarct myocardium of two experimental groups (*n* = 4 per group). (F) qPCR data showing the levels of HADC6 gene in the infarct myocardium of both groups. The significance of differences was assessed by unpaired Student's *t*‐test (C, E, F).

At the indicated time points before and after shame and MI operations, following hemodynamic analysis by an echocardiography (Figure 1A), all mice were anaesthetised with an intraperitoneal injection of chloral hydrate (0.1 mL/10 g), and blood samples were collected from the left ventricles. Following perfusion with 4% phosphate buffered saline (PBS) at the physical pressure, the whole hearts and left ventricles were successively isolated and weighted. For the biological analysis, the LV tissues was maintained in RNA later solution (for the gene assay) or stored at −80°C (for the protein assay). For the morphological analysis, after being immersed in fixative at 4°C, the LV tissues were embedded in optimal cutting temperature compound (Sakura Fine‐technical, Tokyo) and stored at −20°C. The blood was poured into a blood collection tube and centrifuged, and the plasma was collected for the evaluations of cardiac injury biomarkers (i.e. lactate dehydrogenase [LDH], creatine kinase‐MB [CK‐MB] and cardiac troponin I [cTnI]) and stored at −80°C. All surgical and sampling procedures followed were in accordance with institutional guidelines.

### 2.4 Echocardiography

Transthoracic two‐dimensional (2D) parasternal short‐axis M‐mode echocardiography was performed at baseline and at day 14 after MI surgery, using a Vevo 2100 system (VisualSonics, Toronto, Canada) with a 30‐MHz transducer. In brief, the mouse was selected and placed into an anaesthesia induction kit full of 2% isoflurane until the mouse became unconscious. The mouse was then fixed in a supine position on a heating plate (37°–38°C) with its limbs dotted with pieces of adhesive tape. Hair removal cream was applied to remove the hair on the chest area. After the thorax of the mouse was cleaned, the thorax was coated with an ultrasonic coupling agent. When the long‐axis surface of the left ventricle was detected and rotated 90° clockwise, the image of the short axis of the left ventricle appeared. We observed the ventricular wall motion and saved the left ventricular (LV) long‐ and short‐axis views under the B‐mode and M‐mode. The left ventricular ejection fraction (LVEF), LV fraction shortening (LVFS), LV end‐diastolic diameter (LVDd), LV end‐systolic diameter (LVSd) and the mitral valve E peak/A peak ratio (E/A ratio) of the LV inflow were calculated with doppler measurements. The mouse heart rate (HR) was also calculated.

### 2.5 Western blotting

Total proteins were extracted from heart tissue and cardiac fibroblasts as samples for Western blotting.^18^ Samples were homogenized and lysed with RIPA buffer containing protease and phosphatase inhibitor cocktails for 30 min on ice. The lysates were then centrifuged at 13,000 *g* for 10 min, and the supernatants were collected and labelled. The total protein concentrations in the supernatants were measured by a bicinchoninic acid assay (Pierce BCA Protein Assay Kit; Thermo Scientific, Waltham, MA). Twenty micrograms of protein per lane were resolved by SDS‐PAGE, transferred to PVDF membranes (Bio‐Rad, Hercules, CA). Each membrane was blocked with 5% nonfat milk and incubated with primary antibodies overnight at 4°C. On the following day, the membranes were incubated with secondary antibodies (Cell Signaling Technology [CST], Danvers, MA).

Immunoreactive signals were detected using an Azure 500 analyzer (GE Healthcare, Milwaukee, WI). The following antibodies were used for Western blotting: rabbit anti‐phospho‐Smad2/3 (p‐Smad2/3) monoclonal antibody (mAb) (#8828S, 1:1000), rabbit anti‐Smad2/3 mAb (#8685S, 1:1000), rabbit anti‐TGF‐β1 mAb (#3711S, 1:500), anti‐HDAC6 mAb (#7558S, 1:1000), anti‐GAPDH mAb (#5174S, 1:6000), mouse anti‐extracellular signal regulated kinase1/2 (Erk1/2) mAb (#9107, 1;1000) and anti‐p‐Erk1/2^t202/t204^ mAb (#4377, 1:1000) were purchased from Cell Signaling Technology (Danvers, MA). Mouse‐anti‐Nkx‐2.5 mAb (sc‐376565, 1:1000) was purchased from Santa Cruz Biotechnology (Santa Cruz, CA). Rabbit anti‐TGF‐β1 receptor (TGF‐βR1) polyclonal antibody (pAb, #SAB4502958, 1:500) was purchased from Sigma‐Aldrich (St. Louis, MO). Rabbit anti‐Collagen I mAb (#EPR22894‐89, 1:1000) and rabbit anti‐Collagen III mAb (#EPR17673, 1:1000) were purchased from Abcam (Cambridge, MA). Anti‐α‐SMA antibody (#AF1032, 1:1000) was from Affinity Biosciences (Cincinnati, OH).

### 2.6 Real‐time polymerase chain reaction (RT‐PCR) assay

Total RNA was extracted from tissues using Trizol reagents (Invitrogen, Carlsbad, CA), and first‐strand cDNA was synthesized using a Thermo Script RT‐PCR synthesis kit (Fermentas, Burlington, ON, Canada).^19^ Quantitative PCR (qPCR) analyses of mRNA were performed using Thermo Script RT‐PCR kits (Fermentas). The qPCR was carried out under a standard protocol using the following primers. The PCR was performed in triplicate. Each PCR was performed at 95°C for 10 min, followed by 40 cycles of 95°C for 15 s and 65°C for 1 min. The expression of GAPDH was measured in parallel with that of the genes of interest and was used as an internal standard for the quantitative comparison of mRNA levels. The generation of specific genes' expression changes was evaluated using the comparative Ct method, X = Z^−∆∆Ct^. Fold‐alterations were evaluated using the ∆∆Ct method and compared with GAPDH. The sequences of mice targeted gene primers was listed in the Table 1.

**TABLE 1:** Sequences of mice primers and siRNA used for qPCR and targeted gene silencing.

Genes	Forward primers	Reverse primers
qPCR		
Collagen I	AGGCGAAGGCAACAGTCG	GTTCCGGYGTGACTCGTGC
Collagen III	AGGTTCTCCTGGTGCTGCT	GGATGCCCACTTGTTCCAT
HADC6	CGAGTTCTTGCAGGCACCTA	ATGCTCATAGCGGTGGATGG
GAPDH	ATGTGTCCGTCGTGGATCTGA	ATGCCTGCTTCACCACCTTCT
Targeted RNA silencing		
HADC6	GGUAUUUGAUGAACAGCUATT	UAGCUGUUCAUCAAAUACCTT
MAPK1	CCCUCACAAGAGGAUUGAATT	UUCAAUCCUCUUGUGAGGGTT
siRNA‐NC	UUCUCCGAACGUGUCACGUTT	ACGUGACACGUUCGGAGAATT
CY3 siRNA‐NC	UUCUCCGAACGUGUCACGUTT	ACGUGACACGUUCGGAGAATT

Abbreviations: GAPDH, gluceradehyde‐3‐phosphate dehydrogenase; HDAC6, Histone Deacetylase 6; MAPK1, mitogen‐activated protein kinase 1; siRNA NC, short interfering RNA negative control; siRNA: short interfering RNA.

### 2.7 Evans blue and TTC staining

On the 14th day post‐MI or sham operation, mice were injected 2% with Evans Blue dye (Sbjbio Life Sciences, Nanjing, China) via the caudal vein, and their hearts were excised and frozen at −80°C. Fifteen minutes later, the hearts were minced into approximately 1‐mm‐thick slices and submerged in a 1% triphenyl tetrazolium chloride (TTC) (Solarbio Science & Technology Co., Beijing, China) solution at 37°C for approximately 15 min. Healthy areas of a mouse heart are stained blue; the area at risk (AAR) turns red, and the infarct area (IA) remains unstained as white. To determine the sizes of the AAR and IA, we calculated the ratio between the IA and AAR with image analysis software (Image J).

### 2.8 Histology and collagen deposition evaluation

Masson's trichrome staining was performed as described.^20^ After mice were sacrificed, cardiac tissue was sectioned (5–6 μm) from below the ligation plane of the heart toward the apex with the use of a cryostat (CM1950, Leica Biosystems, Wetzlar, Germany) and dehydrated in 20% sucrose solution. The slices were fixed in 4% polyformaldehyde for 24 h and then embedded in OCT. At first, transverse tissue sections were stained with haematoxylin and eosin (H&E) and the cross‐sectional area of myocytes was determined from cells that were cut transversely and exhibited both a nucleus and an intact cell membrane; at least 100 cells were assessed per specimen, and the average value was used for analysis (×200). And transverse tissue sections were stained with Masson's trichrome. Sections were imaged using an EVOS FL Auto 2 imaging system (Thermo Fisher Scientific). To determine the extents of interstitial fibrosis in the infarct area, we selected five fields at random and calculated the ratio of the area of Masson's trichrome‐stained fibrosis to the total area of the left ventricles with the Image J software.

### 2.9 Immunofluorescence

The immunofluorescence assay was performed as described.^21^ Following blocking with bovine serum albumin for 30 min, the LV tissue sections and the cultured cells (cover‐glasses) were treated with the primary antibodies to HDAC6, TGF‐β1, TGF‐βR1, α‐actin, vimentin or CD206 (1:250 for each antibody), respectively, overnight. After being washed with PBS for three times, the tissue sections and the cells were treated with the fluorescent labelled secondary antibodies against anti‐rabbit IgG or goat anti‐mouse IgG (1:200 for each), respectively, for 1 h at room temperature, and then the nuclei were counterstained with an anti‐fluorescence quenching solution including DAPI. Sections were imaged using an EVOS FL Auto 2 imaging system (Thermo Fisher Scientific). The levels of HDAC6 and CD206 protein expressions in the LV tissues were evaluated in four random microscopic fields from three independent sections of each animal (*n* = 5) and were expressed as the percentages of the positive staining signal intensities per high‐power field (×400). And the levels of TGF‐β1 and its receptor TGF‐βR1 protein expressions of the targeted cells were evaluated in two‐three random microscopic fields from three independent sections of each cell seeded cover‐glass (*n* = 5) and were expressed as the numbers of the positive staining cells per high‐power field (×400).

The following commercially available antibodies were used for the immunofluorescence: Mouse anti‐α‐actinin mAb (#sc‐17829) and anti‐Vimentin mAb (#sc‐6260) were purchased from Santa Cruz Biotechnology. Rabbit anti‐TGF‐βR1 pAb (#SAB4502958) was purchased from Sigma‐Aldrich (Burlington, MA). Rabbit anti‐TGF‐β1 mAb (#ab315254) was purchased from Abcam (Cambridge, MA). Rabbit anti‐HDAC6 mAb (#7558S) was from Cell Signaling Technology. And the secondary goat anti‐rabbit IgG pAb (FITC 495) and anti‐mouse IgG pAb (Alexa Fluor 594) were purchased from APExBIO (Houston, TX).

### 2.10 Cardiac fibroblast (CF) culturing

Ethylenediaminetetraacetic acid (EDTA) (0.5%) was used to digest detached cardiac fibroblasts (CFs) (Cat no. CPR‐074; Procell Life Science & Technology Co., Wuhan, China), and the supernatant was collected in 10‐mL Falcon centrifuge tubes. Four of these tubes were then centrifuged to collect the supernatant. The dispersed cells were resuspended in six‐well plates containing 6 mL of Dulbecco's modified Eagle's medium (DMEM) with 15% fetal bovine serum (FBS), 1% penicillin and streptomycin. Two hours later, the plates were washed with warmed phosphate‐buffered saline (PBS) and replenished with cell culture medium.

The CFs were then cultured until they reached approx. 80%–90% confluence. After being cultured in serum‐free DMEM for 6 h, the CFs were treated with Tub A at the indicated concentrations (0, 5 and 10 μmol/L) under normoxic (5% CO_2_ and 95% air) or hypoxic conditions (1% O_2_, 5% CO_2_ and 94% N_2_), respectively, for 24 h and then subjected to biological analyses.

### 2.11 Cell transfection

Cell transfection was performed as described.^22^ CFs were seeded on six‐well plates and cultured for until they were 50%–60% sub‐confluent. The cells were then transfected with short interfering (si)RNA against HDAC6 (siHDAC6, 100 nmol/L) or non‐targeting control siRNA (NC, both from Hanheng Biotechnology Co., Shanghai, China) using Lipofectamine 3000 (Thermo Fisher Scientific). After transfection for 48 h, the cells were cultured in normoxic or hypoxic conditions (1% O_2_, 5% CO_2_ and 94% N_2_), respectively, for 24 h and then subjected to a Western blotting assay. The siRNA sequences are listed in Table 1.

### 2.12 Statistical analysis

All data are expressed as the mean ± standard error of the mean (SEM). We performed a one‐way analysis of variance (ANOVA) for comparisons of multiple groups, followed by Tukey's post hoc test or by Student's *t*‐test for comparisons of two independent sample groups with Prism 9.0 software. After we determined the status of the data distribution, the data were subjected to the statistical analysis. If the homogeneity of variance assumption was violated, the nonparametric Kruskal–Wallis test was used instead of Pearson's chi‐square or Fisher's exact test. Survival rate was analysed by the standard Kaplan–Meier method with a log‐rank test. Morphological and histological characteristics were evaluated by two observers in a blind manner, and the values they obtained were averaged. Probability (*p*)‐values <0.05 were considered significant.

## 3 RESULTS

### 3.1 The post‐MI changes in HDAC6 expression

To study the impact of the ischemic stress on HDAC6 expression, we randomly assigned HDAC6^+/+^ mice to the sham operation and LAD ligation surgery and subjected the mice to morphological and biological analyses at the indicated time points (Figure 1A). The HR and LVEF were tested to evaluate the success of the model (Figure 1B–D). The results of the qPCR and Western blotting assays demonstrated that the expression level of HDAC6 was significantly increased in the LV infarct myocardium of MI group compared to the control group (Figure 1E–G), indicating that HDAC6 is likely to participate in the cardiac pathological remodelling in the post‐MI phase of the murine MI model.

### 3.2 HDAC6 deletion prevented cardiac fibrosis and remodelling after MI

**FIGURE 2:**
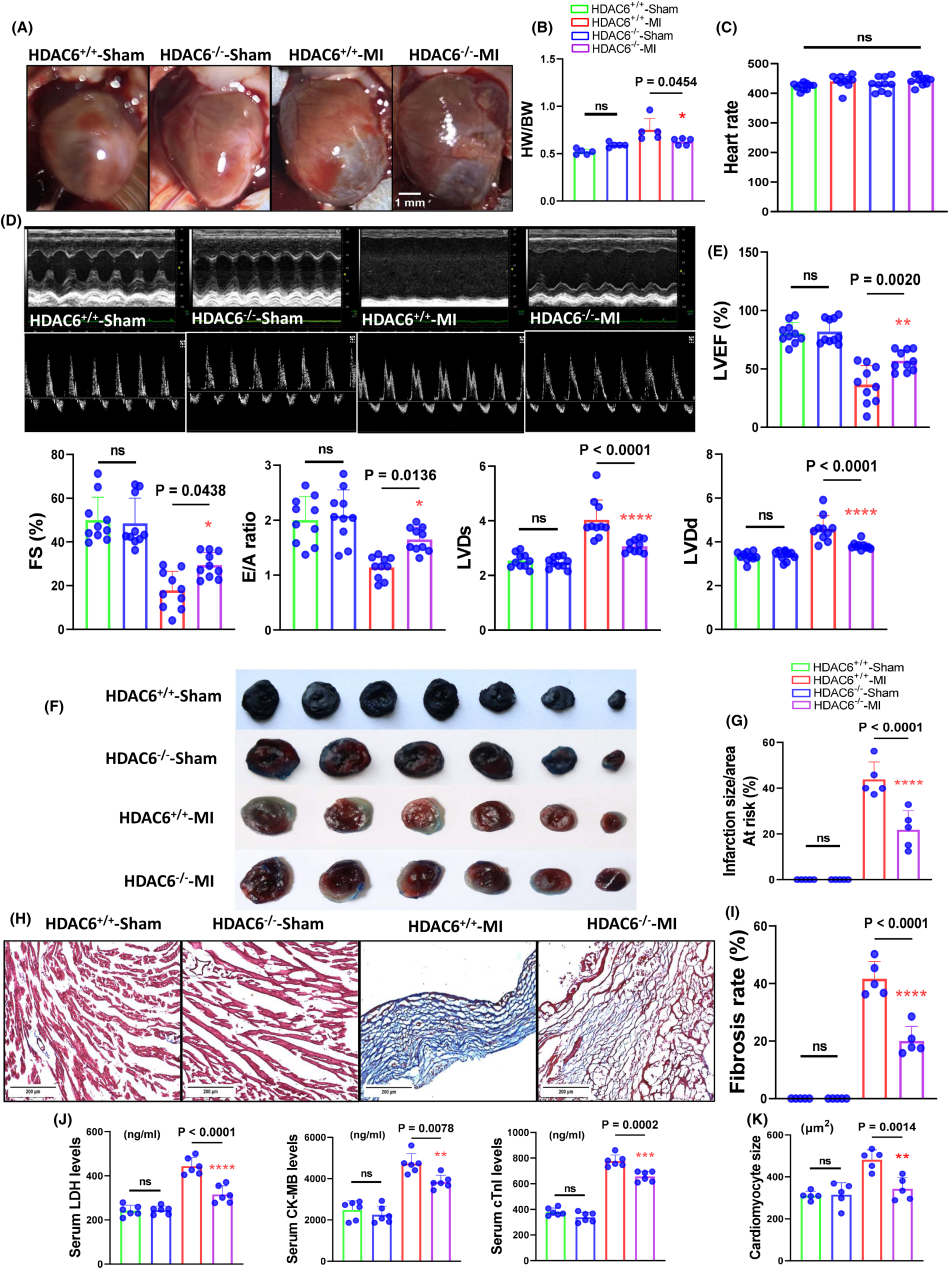
HDAC6 deletion ameliorated the cardiac infarct size and remodelling in the post‐MI phase. Eight‐week‐old male HDAC6^+/+^ and HDAC6^−/−^ mice were randomly assigned to the sham operation (HDAC6^+/+^‐Sham and HDAC6^−/−^‐Sham) or MI surgery (HDAC6^+/+^‐MI and HDAC6^−/−^‐MI) and subjected to echocardiography analyses at day 14 after surgery. (A) Representative images of the heart view in the four groups. (B) The quantitative data showing the heart weight (HW)/body weight (BW) ratio in the four experimental groups (*n* = 5 per group). (C) Heart rate (HR) showing for four experimental groups (*n* = 10 per group). (D, E) Representative echocardiographic images and combined quantitative data showing the LVEF, LVFS, LVDd, LVDs and E/A ratio (the ratio of the mitral E‐peak to the A‐peak) in the four experimental groups (*n* = 10 per group). (F, G) Representative images and quantitative data of the Evans blue & TTC staining analyses for the percentage of the infarct size to the risk area in the four groups (*n* = 5 per group). (H, I) Representative Masson's trichrome staining images and quantitative data show the percentage of fibrosis area in the four groups (*n* = 5 per group). (J) Quantitative data of the H&E staining show the cardiomyocyte size in the three groups (*n* = 5 per group). (K) The ELISA data show the levels of serum LDH, cTnI and CK‐MB in the four groups (*n* = 6 per group). The significance of differences was assessed by a one‐way ANOVA with Tukey's post hoc tests in panels B, C, E, G, I and J. NS, not significant.

Figure 2A provides representative heart photos from the four experimental groups at day 14 after the ligation or sham operation. We observed that the heart weight (HW)/body weight (BW) ratio was lower in the HDAC6^−/−^‐MI mice compared to the HDAC6^+/+^‐MI mice (Figure 2B). As shown in Figure 2C, there was no difference in HR between both genetic MI groups. The quantitative echocardiography data revealed that compared to the HDAC6^+/+^‐MI mice, HDAC deficiency significantly ameliorated the reductions of the LVEF, LVFS, LVLd, LVDs and E/A ratio values in the post‐MI mice (Figure 2D,E). Consistently, HDAC6 deletion markedly lowered the infarct size and cardiomyocyte size and reduced the degree of fibrosis as well as the levels of serum LDH, cTnI and CK‐BM (Figure 2F–K), suggesting that HDAC6 deficiency was resistant to ischemic injury in mice. However, there were no significant differences in these nine parameters (LDH, cTnI, CK‐BM, HW/BW ratio, HR, LVEF, LVFS, LVLd, LVDs, E/A ratio, infarct size and fibrosis area) between the sham‐operated mice of both genotypes (Figure 2).

**FIGURE 3:**
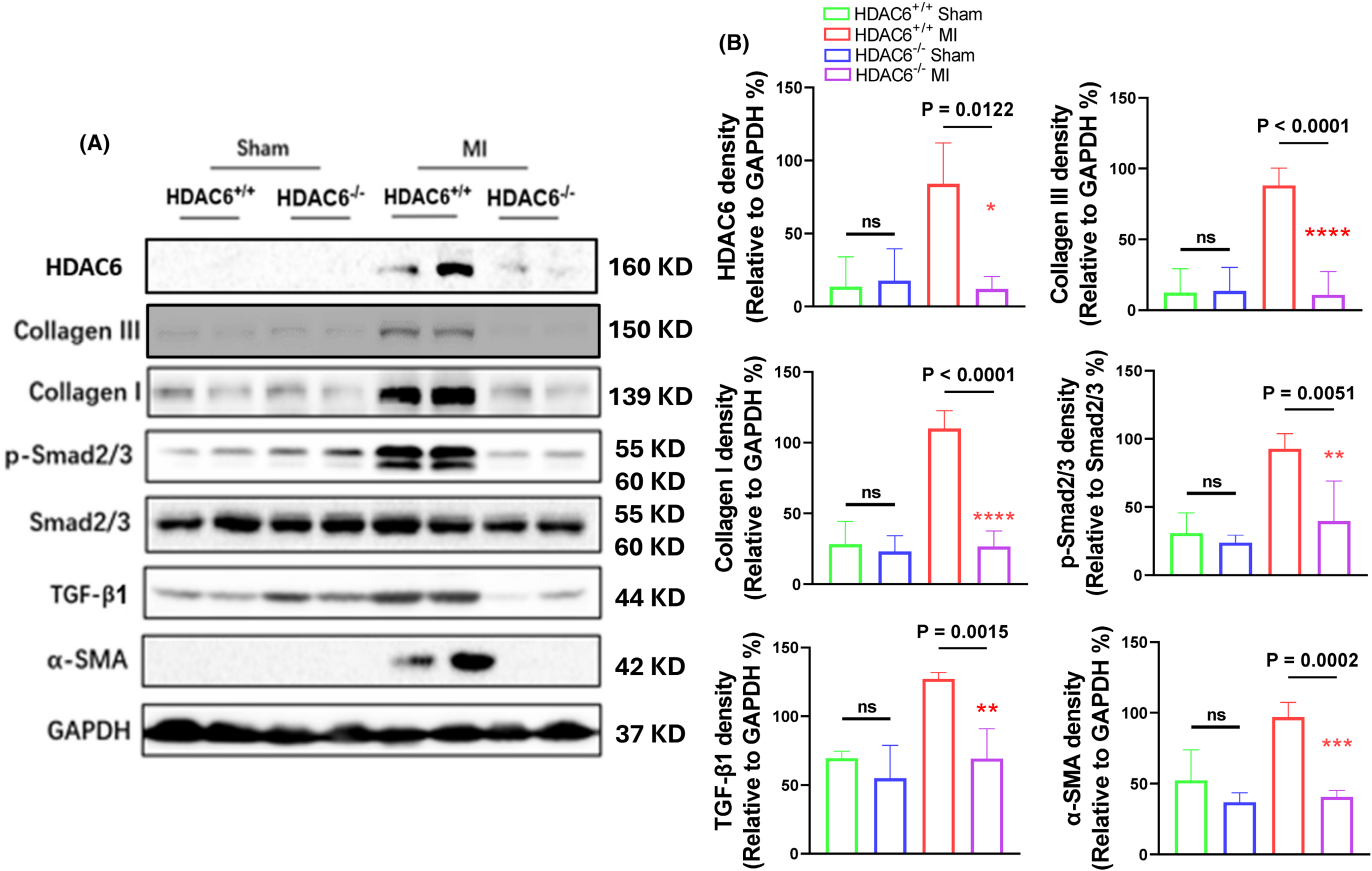
HDAC6 deletion lowered the TGF‐β1/Smad2/3 signalling activation that occurred in response to ischemic stress at day 14 after surgery. (A, B) Representative immunoblotting images and combined quantitative data showing the levels of collagen I, collagen III, p‐Smad2/3, TGF‐β1 and α‐SMA in the infarct myocardium of the four mouse groups (*n* = 4 per group). The significance of differences was assessed by a one‐way ANOVA with Tukey's post hoc tests in panel B. NS, not significant.

**FIGURE 4:**
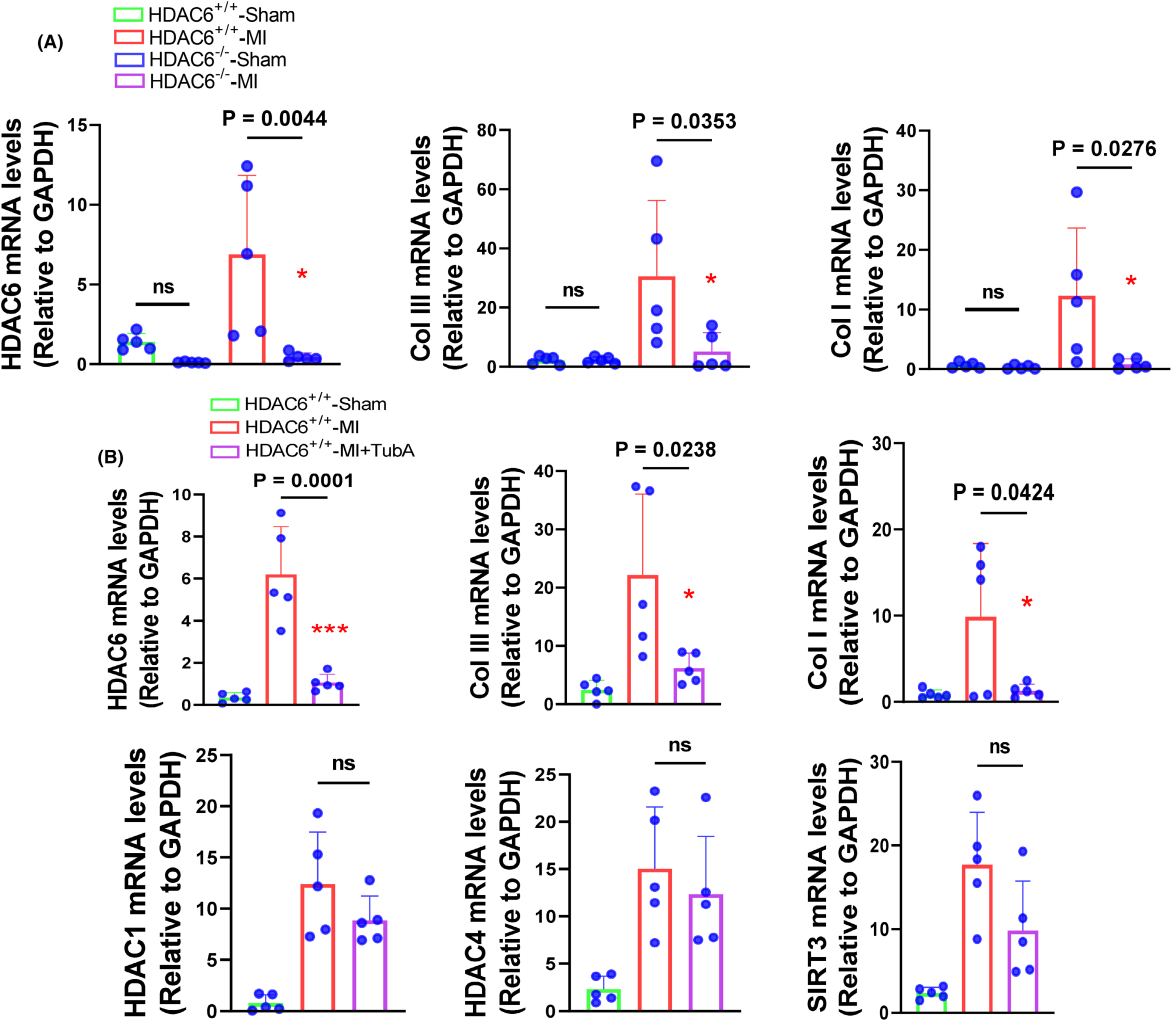
Genetic and pharmacological inhibitions of HDAC6 lowered the targeted molecule gene expressions in the LV myocardium in response to ischemic stress. (A) qPCR data showing that the levels of collagen I and III mRNAs in four experimental groups (HDAC6^+/+^‐Sham, HDAC6^−/−^‐Sham, HDAC6^+/+^‐MI and HDAC6^−/−^‐MI) (*n* = 5 per group). (B) qPCR data showing that the levels of HDAC1, SIRT3, HDAC4, HADC6 and collagens I and III mRNAs in three experimental groups (HDAC6^+/+^‐Sham, HDAC6^+/+^‐MI and HDAC6^+/+^‐TubA) (*n* = 5 per group). In panels A and B, significance was assessed by a one‐way ANOVA with Tukey's post hoc tests. NS, not significant.

To explore the molecular mechanisms of HDAC6^−/−^‐mediated cardioprotection in the post‐MI phase, we conducted a Western blotting assay to focus on the TGF‐β1/Smad2/3 signalling pathway. As anticipated, the HDAC6^−/−^‐MI mice exhibited significant reductions in the levels of p‐Samd2/3, TGF‐β1, alpha‐smooth muscle action (α‐SMA) and collagen I and III proteins compared to the HDAC6^+/+^‐MI mice (Figure 3A,B). The qPCR yielded the same conclusions for the collagen I and III gene expressions (Figure 4A). HDAC6 thus appeared to modulate cardiac remodelling and dysfunction through the modulation of TGF‐β1/Samd2/3 signalling pathway activation in the post‐MI phase.

### 3.3 HDAC6 inhibition produced cardiac benefits after MI

**FIGURE 5:**
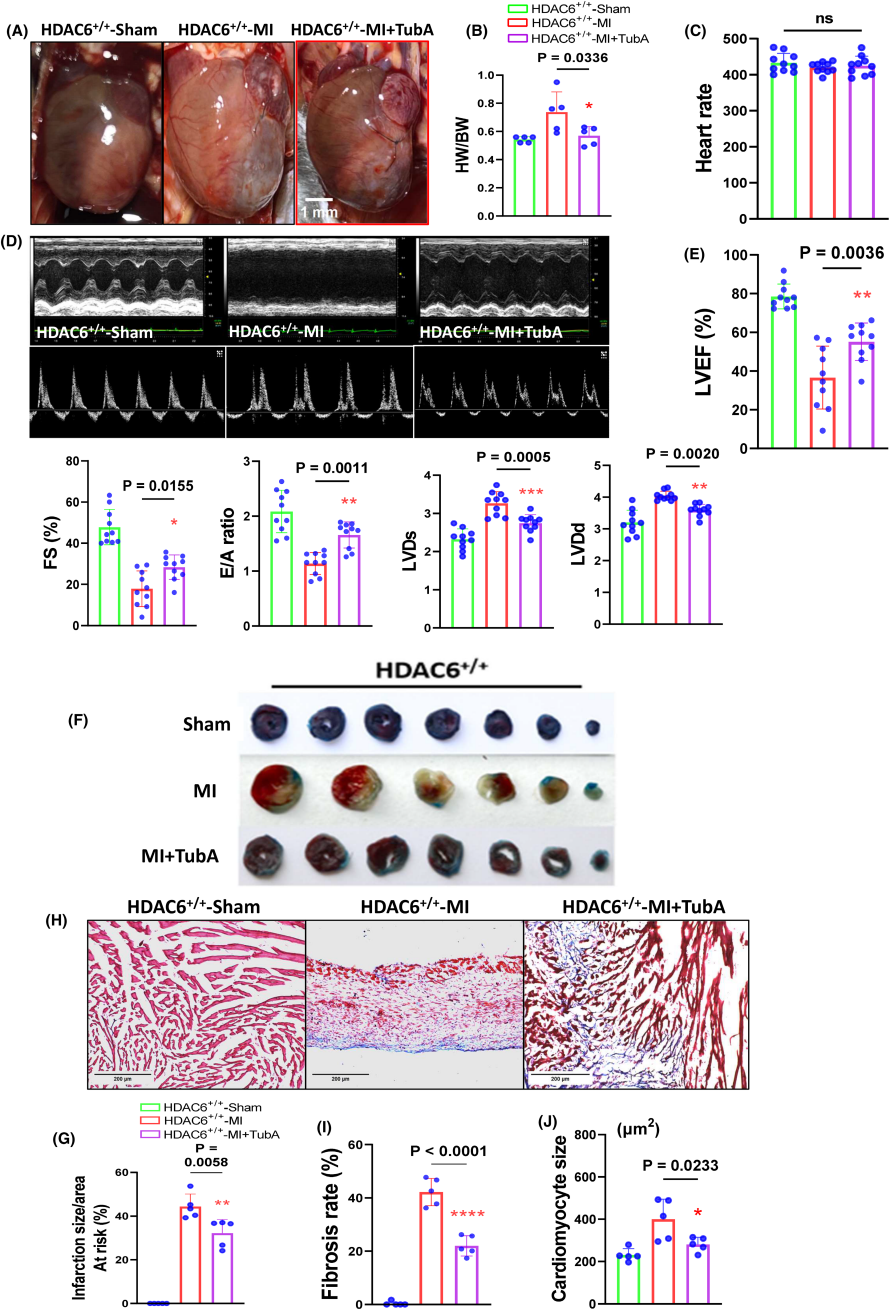
HADC6 inhibition also ameliorated the myocardial infarct size and dysfunction in the post‐MI state. Eight‐week‐old HDAC6^+/+^ mice underwent a sham operation and received vehicle (HDAC6^+/+^‐Sham), MI surgery plus vehicle (HDAC6^+/+^‐MI), or MI surgery plus tubastatin A (TubA, HDAC6^+/+^‐TubA; 10 mg/kg/day) for 14 days. (A) Representative images of the heart in the three groups. (B) The quantitative data show the HW/BW ratio of the experimental groups (*n* = 5 per group). (C) Heart rate (HR) showing for four experimental groups (*n* = 10 per group). (D, E) Representative echocardiographic images and combined quantitative data showing the levels of LVEF, LVFS and the E/A ratio (*n* = 10 per group). (F, G) Representative images and quantitative data of the Evans blue & TTC staining analyses showing the percentage of infarct size to the risk area in the three groups (*n* = 5 per group). (H, I) Representative Masson's trichrome staining images and quantitative data show the percentage of fibrosis area in the three groups. (J) Quantitative data of the H&E staining show the cardiomyocyte size in the three groups (*n* = 5 per group). Statistical significance was assessed by a one‐way ANOVA with Tukey's post hoc tests in panels B, D, F and H.

**FIGURE 6:**
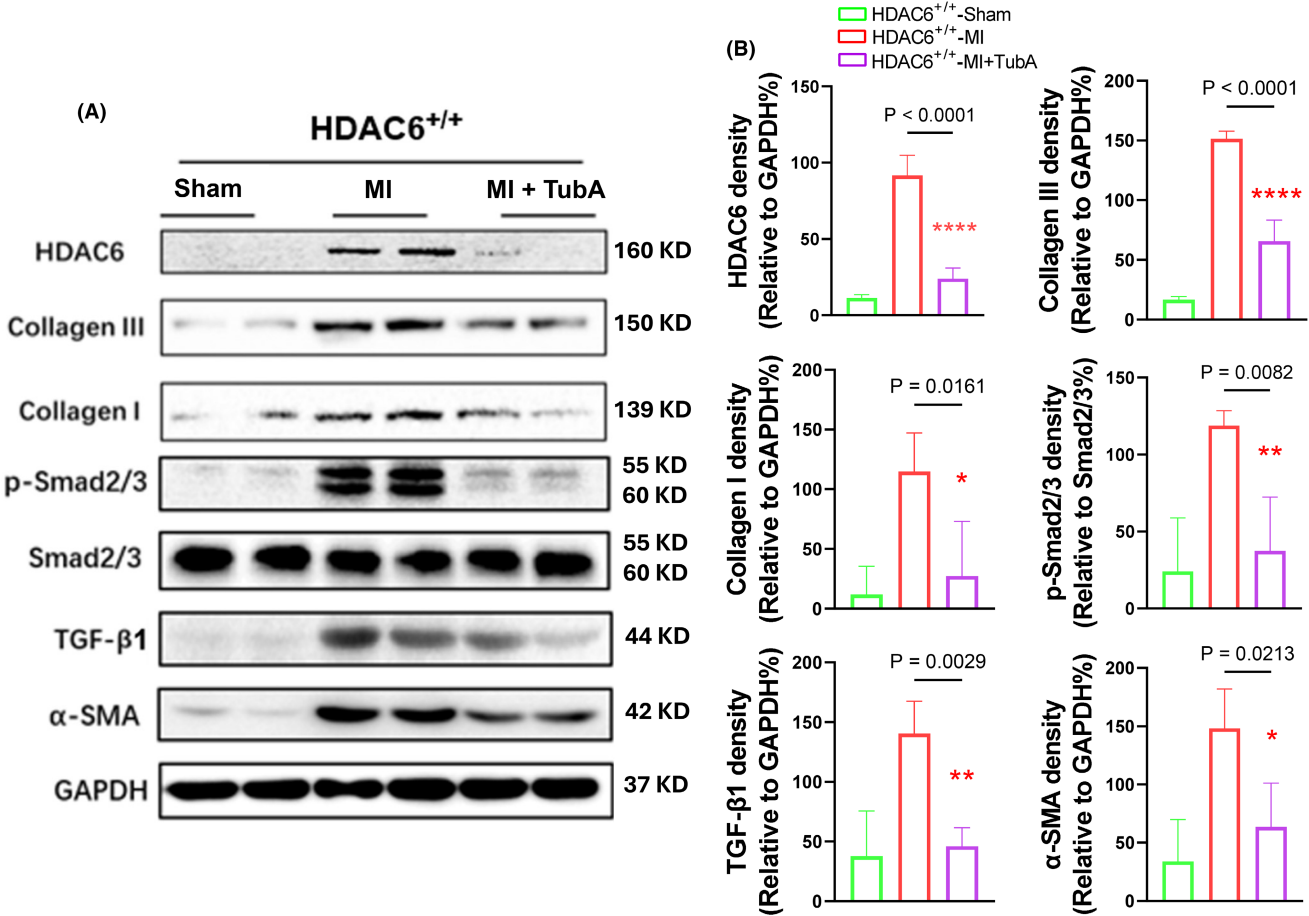
HDAC6 inhibition lowered the TGF‐β1/Smad2/3 signalling activation that occurred in response to ischemic stress. (A, B) Representative immunoblotting images and combined quantitative data showing the levels of HDAC6, collagen I, collagen III, p‐Smad2/3, smad2/3, TGF‐β1 and α‐SMA in the LV myocardium of the three experimental groups. In panel B, significance was assessed by a one‐way ANOVA with Tukey's post hoc tests.

**FIGURE 7:**
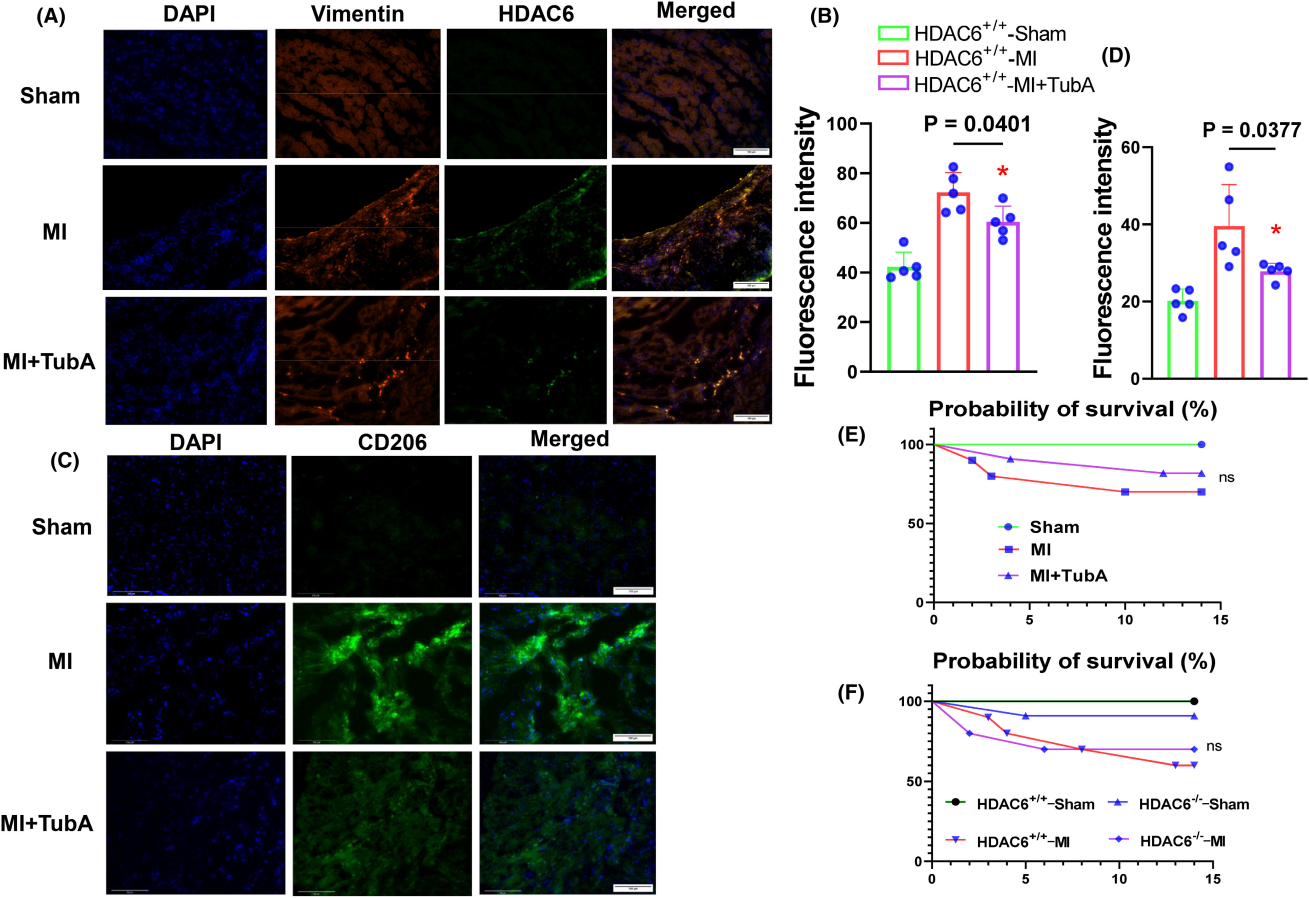
HDAC6 inhibition lowered ischemia‐induced cardiac HDAC6 and CD206 protein expressions. (A–D) Representative immunofluorescence image and combined quantitative data show the levels of HDAC6 and CD206 proteins in the LV myocardium of three experimental groups (*n* = 5, each group). (E, F) Kaplan–Meier plots of the survival rates of mice for three or four experimental groups (*n* = 10). Statistical significance was assessed by a one‐way ANOVA with Tukey's post hoc tests for Figure B and D or the standard Kaplan–Meier method with a log‐rank test for E and F. NS, not significant.

Figure 5A provides representative heart photos from the three experimental groups at day 14 after the ligation or sham operation. As shown in Figure 5B, the TubA treatment also markedly reduced the ratio of HW to BW of the HDAC6^+/+^‐MI mice. We observed that in the HDAC6^+/+^‐MI mice, TubA loading resulted in reductions in the values of HR, LVEF, LVFS, LVDd, LVDs and the E/A ratio as well as the cardiac infarct size and cardiomyocyte size as well as collagen accumulation (Figure 5C–J). The quantitative data of the Western blotting images demonstrated that the protein levels of HDAC6, p‐Samd2/3, TGF‐β1, α‐SMA, collagen I and collagen III were markedly lower in the LV myocardium of the HDAC6^+/+^‐TubA MI mice compared to the control non‐treated HDAC6^+/+^‐MI mice (Figure 6). The qPCR yielded same conclusions regarding collagen I and III as well as HDAC6 gene expressions (Figure 4B). Immunofluorescent analysis revealed only a low positive staining signal of HDAC6 in the LV myocardium of control mice (Figure 7A,B). And the staining signal of HDAC6 was markedly increased in the infarcted myocardium of mice with AMI, with staining apparent in CFs and these changes were reduced by TubA treatment (Figure 7A,B). Similarly, the HDAC6^+/+^‐Tub A MI mice had decreased levels of CD206 protein expression as compared to the control non‐treated HDAC6^+/+^‐MI mice (Figure 7C,D). Collectively, these observations suggest that the up‐regulation of HDAC6 by ischemic injury could act a key modulator of cardiac response of post‐MI mice. However, we observed that pharmacological and genetic interventions targeted toward DAC6 had no effect on mice mortality and/or the levels of HADC1, SIRT3 and HADC4 genes in the infarcted myocardium (Figures 4B,7E,F).

### 3.4 HDAC6 silencing lowered the CF collagen production

**FIGURE 8:**
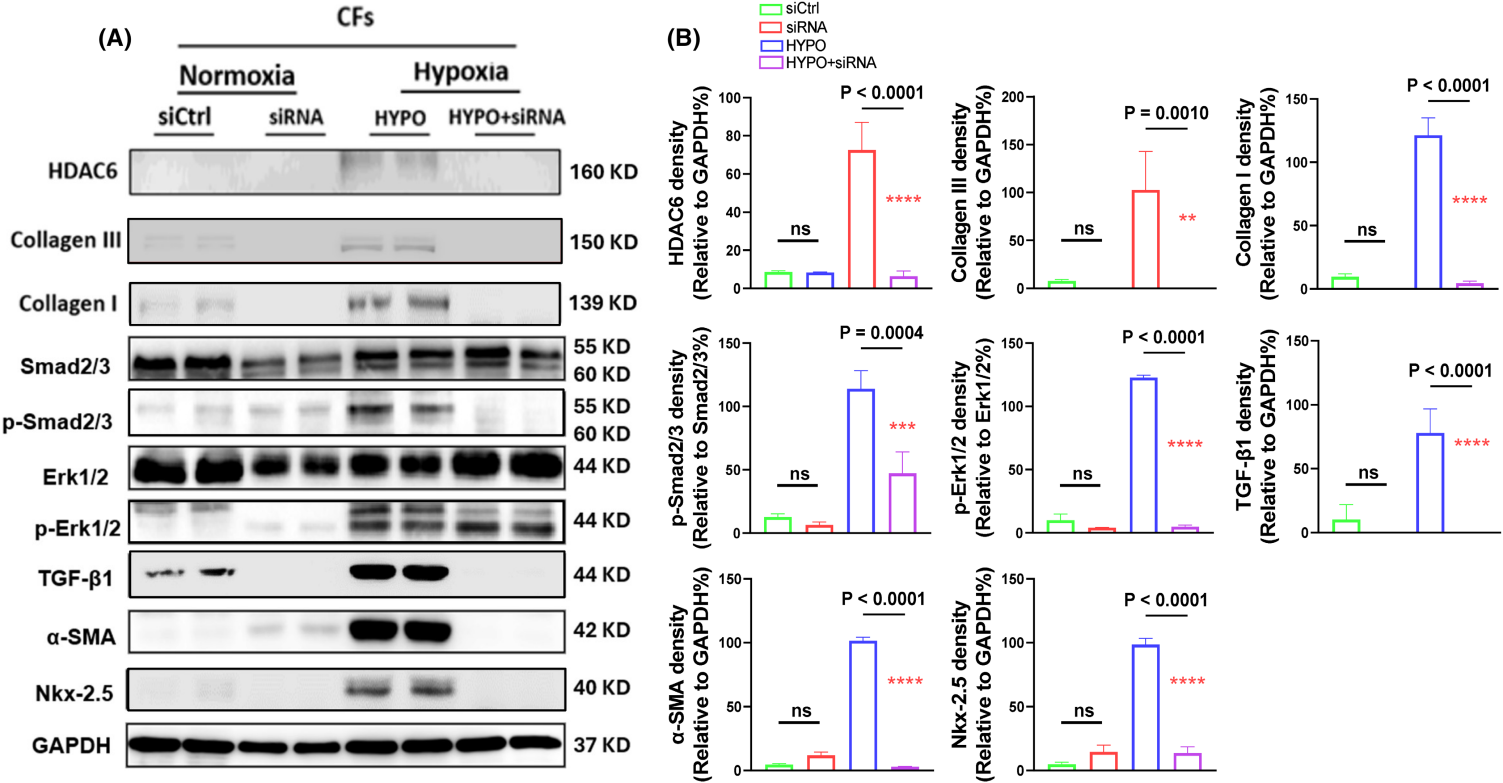
HADC6 silencing reduced the levels of the targeted proteins in cultured cardiac fibroblasts (CFs) in response to hypoxia. Following transfection with control non‐targeting short interfering RNA (siCtrl) and targeted siRNA against HADC6, the cells were cultured in normoxic (5% CO_2_ and 95% air) or hypoxic (5% CO_2_ and 95% air) conditions respectively for 24 h and the lysates were used in a Western blotting assay. (A, B) Representative Western blotting images and quantitative data showing the levels of HADC6, collagen I, collagen III, p‐Smad2/3, p‐Erk1/2, α‐SMA and Nkx‐2.5 proteins in the four groups (*n* = 4 for each group). In panels B, significance was assessed by a one‐way ANOVA with Tukey's post hoc tests. NS, not significant.

**FIGURE 9:**
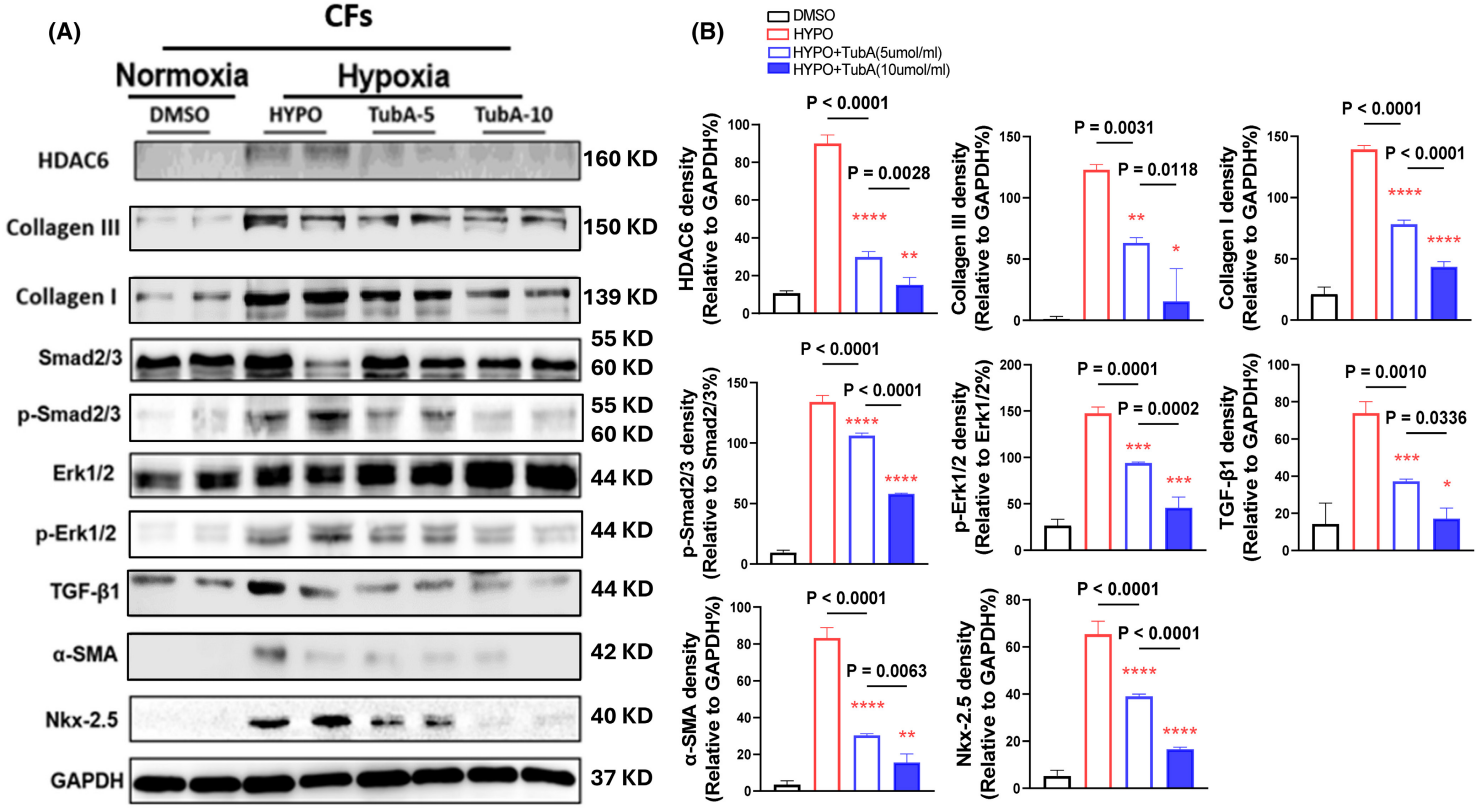
HADC6 inhibition reduced the levels of the targeted proteins in cultured cardiac fibroblasts (CFs) in response to hypoxia. Following serum‐free culturing for 6 h, the CFs were treated with the indicated TubA concentration under normoxic (5% CO_2_ and 95% air) or hypoxic (5% CO_2_ and 95% air) conditions respectively for 24 h, and the lysates were subjected to a Western blotting assay. (A, B) Representative Western blotting images and quantitative data showing the levels of HDAC6, collagen I, collagen III, p‐Smad2/3, p‐Erk1/2, α‐SMA and Nkx‐2.5, proteins in the groups (*n* = 4, each group). In panels B, significance was assessed by a one‐way ANOVA with Tukey's post hoc tests.

**FIGURE 10:**
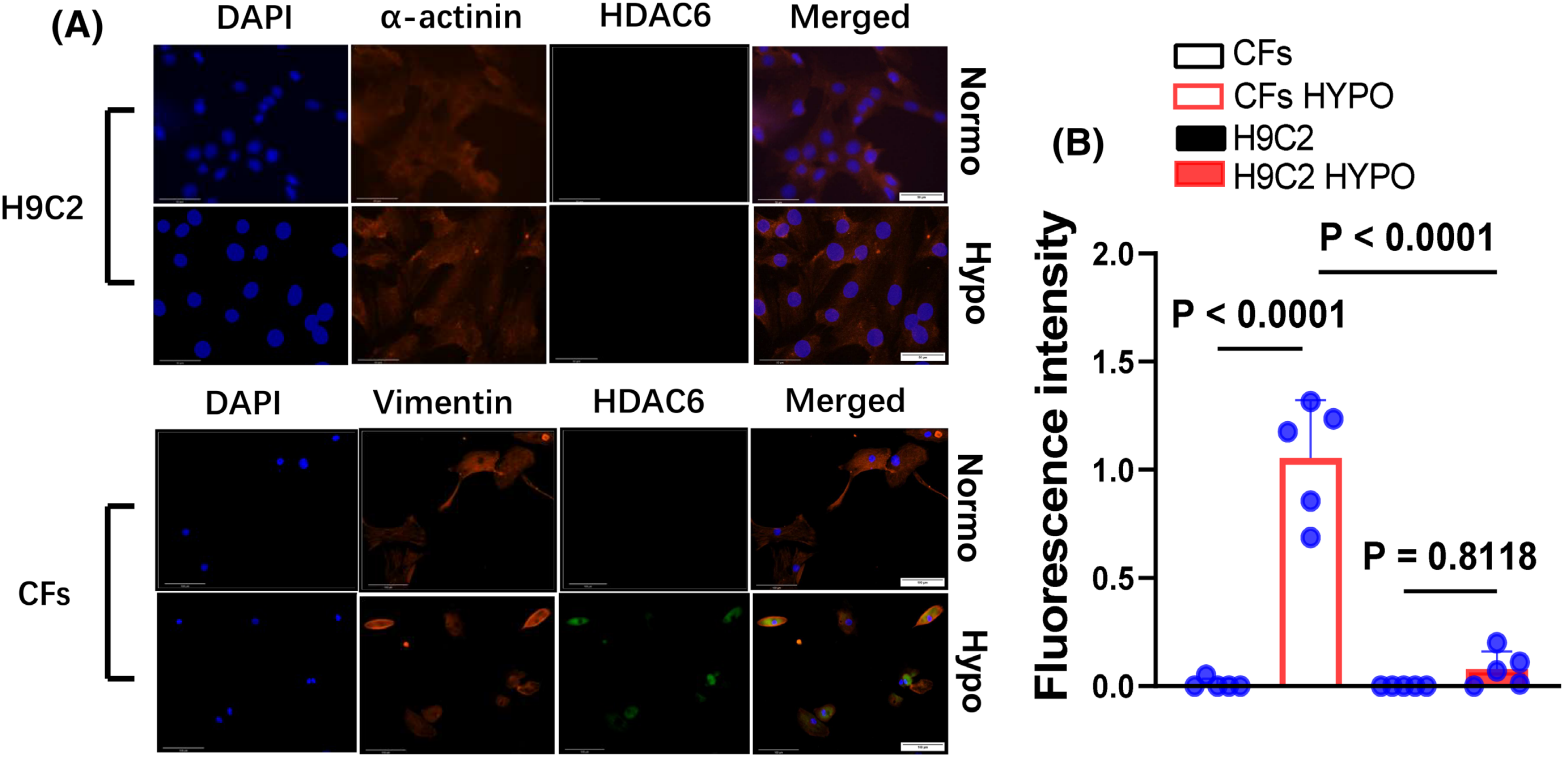
Hypoxia increased HDAC6 protein expression in CFs but not in H9C2. Following treatment in normoxic or hypoxic conditions, respectively, for 24 h, the lysates were used in a Western blotting assay. (A, B) Representative immunofluorescence images and quantitative data show the immunofluorescence signal intensities of HDAC6 protein in cultured H9C2 and CFs under hypoxic conditions (*n* = 5, each group). In panel B, significance was assessed by a one‐way ANOVA with Tukey's post hoc tests. Scar bar: 50 μm (H9C2); 100 μm (CFs).

**FIGURE 11:**
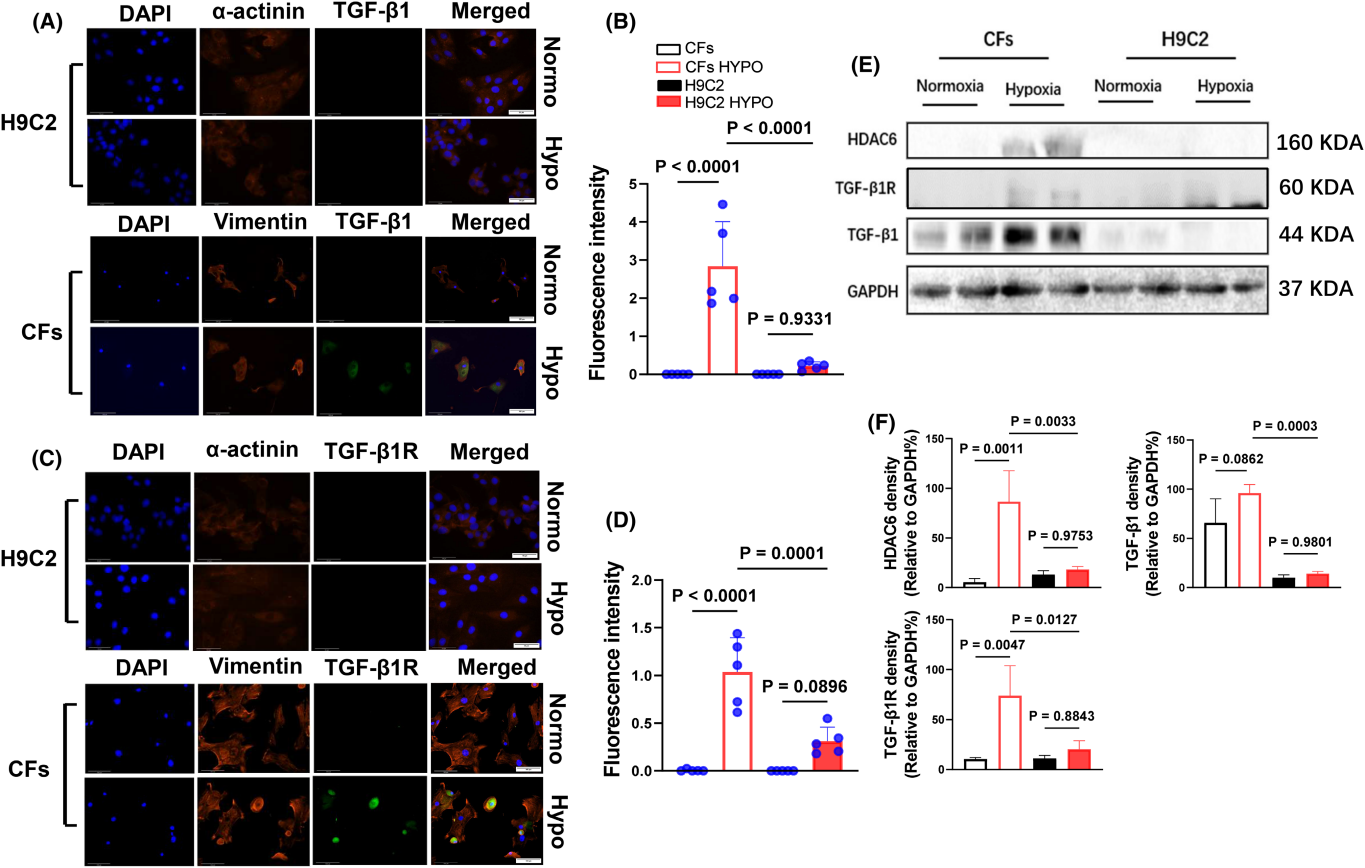
Hypoxia increased the levels of TGF‐β1 and TGF‐βR1 proteins in CFs but not in H9C2. Following treatment in normoxic or hypoxic conditions respectively for 24 h, the lysates were used in a Western blotting assay. (A–D) Representative immunofluorescence images and quantitative data show the immunofluorescence signal intensities of TGF‐β1 and TGF‐βR1 proteins in cultured H9C2 and CFs under hypoxic conditions (*n* = 5, each group). (E, F) Representative Western blots and quantitative data show the levels of TGF‐β1 and TGF‐βR1 proteins in cultured H9C2 and CFs under hypoxic conditions (*n* = 4, each group). Significance was assessed by a one‐way ANOVA with Tukey's post hoc tests for Figure 11B,D,F. Scar bar: 50 μm (H9C2); 100 μm (CFs).

To test our experimental results at the cellular level, we performed an in vitro experiment, and Western blotting was then performed in two different sets of the experiments. The hypoxic condition increased the expressions of HDAC6, p‐Smad2/3, p‐Erk1/2, TGF‐β1, α‐SMA, Nκx‐2.5 and collagen types I and III proteins, and these changes were rectified by HDAC6 silencing and TubA treatment in a dose‐dependent manor, providing evidence of HDAC6‐mediated CF collagen synthesis in response to ischemic stress (Figures 8 and 9). The data of immunofluorescence and Western blotting analysis revealed that hypoxic condition also increased the expressions of HADC6, TGF‐β1 and TGF‐βR1 proteins in CFs, whereas it exhibited a limited effect on these molecular expressions in H9C2 (Figures 10 and 11).

## 4 DISCUSSION

In this study, we focused on novel role(s) of HDAC6 in cardiac remodelling and dysfunction after MI. The ischemic injury elevated HADC6 gene and protein expressions in the injured myocardium of post‐MI mice. The most significant finding of this investigation is that the mice lacking HADC6 were resistant to ischemic injury‐induced cardiac fibrosis and dysfunction. HADC6 deficiency was observed to prevent harmful changes as follows: (*i*) it increased the infarct size and fibrosis area; (*ii*) it impaired indices of LV function (LVEF, LVFS and E/A); and (*iii*) it increased the levels of TGF‐β1, p‐Smad2/3, α‐SMA and collagen I and III proteins and/or genes in the injured myocardium. All of these beneficial effects were reproduced by a pharmacological inhibition of HADC6 in vivo. In vitro, hypoxic stress increased the expressions of HADC6, TGF‐β1, TGF‐βR1, Nkx‐2.5 and collagen I and III proteins or/and genes in CFs but not in H9C2; these alterations were significantly prevented by the HADC6 silencing and TubA loading, providing evidence and a mechanistic explanation for the participation of HADC6 mediated TGF‐β1/TGF‐βR1‐Smad2/3/Nkx‐2.5 and ‐Erk1/2 signalling in cardiac fibrosis and dysfunction in post‐MI mice.

The ability of ischemic injury to enhance the expression of HADC6 probably contributed to the cardiac remodelling and dysfunction in the post‐MI mice. A comprehensive review documented the many roles of the HDAC family in development and physiology, thus providing implications for disease and therapy.^8^ One of our earlier studies demonstrated that HADC6 activity controls injury‐related neointimal hyperplasia via the modulation of a toll‐like receptor‐2‐mediated p38‐mitogen‐activated protein kinase and phosphatidylinositol 3‐kinase/Akt signalling pathway in mice.^23^ In another investigation, the absence of HDAC6 was observed to prevent the development of cardiac dysfunction in hypertrophic and fibrotic conditions.^14^ A single study reported that inhibiting HDAC6 with tubastatin A protected heart function.^24^ We have shown that ischemic stress elevated the HDC6 gene and protein expressions in the infarcted myocardium. Hypoxic stress also resulted in the elevation of HDAC6 protein in CFs not in H9C2, indicating that HDAC6 was sensitive to hypoxic stress in vivo and in vitro of CFs. Our present analyses revealed that HDAC6 deletion improved the cardiac infarct size as well as the impaired LVEF, LVFS and E/A ratio in post‐MI mice. HADC6 inhibition by TubA yielded the same conclusions in the HDAC6^+/+^‐MI mice. Because HDAC6 modulates the myofibril stiffness and diastolic function of the heart, we propose that HDAC6 functioned as an important mediator of ischemia‐induced cardiac dysfunction in mice under our experimental conditions.

TGF‐β1 is synthesized by CFs as in autocrine and paracrine manner under pathophysiological conditions. And TGF‐β1 is secreted by the cardiac cells in an inactive form stored in the extracellular matrix space as a 290‐kDa complex containing the mature homodimer TGF‐β1 (25 kDa), the latent TFG‐β1 binding protein‐1 (190 kDa) and latency‐associated peptide (75 kDa).^25^ Cysteine proteases (i.e. cathepsins B and L) can release an intracellular inactive form of TGF‐β1 (50 kDa).^26^ TGF‐β1‐mediated Smad2/3 signalling has been shown to participate in the cardiac remodelling that occurs in response to ischemic injury in animals.^27^ The protein programmed cell death 5 (PDCD5) was reported to be upregulated by Smad3 during cardiac fibrosis, which subsequently ameliorated progressive fibrosis and cardiac dysfunction through HDAC3 inhibition.^28^ In the present study, HADC6 deletion resulted in a reduction in the levels of TGF‐β1 and p‐Smad2/3 proteins in the LV tissues of post‐MI mice. Interestingly, the TubA‐mediated HADC6 inhibition exerted a beneficial effect on the levels of these targeted proteins in HDAC6^+/+^‐MI mice. Our observations here show that genetic and pharmacological interventions targeted toward HDAC6 ameliorated the elevated levels of TGF‐β1, p‐Smad2/3, Nkx‐2.5 and p‐Erk1/2 in CFs. Hypoxic stress increased the HDAC6 protein expression as well asl TGF‐β1 and its receptor TGF‐βR1 protein expressions in CFs not in H9C2. TGF‐β1 signalling was observed to promote myofibroblast activation and matrix stiffening in a 3D model of human cardiac fibrosis.^29^ Several studies have demonstrated that activated cardiac fibroblast may transdifferentiate into cardiomyoctes.^30^, ^31^ Maioli and colleagues has demonstrated that Smad/Nkx‐2.5 signalling activation is involved in cardiogenesis.^32^ Targeted deletion of Erk2 of cardiomyocytes has been shown to attenuates hypertrophic response in a mouse hypertrophic model.^33^ Moreover, sodium butyrate may exert anti‐hypertrophic effect by suppressing Erk1/2 phosphorylation and HDAC6 activity.^34^ Collectively, these observations suggest that HADC6 may act as a key mediator of TGF‐β1/TGF‐βR1‐mediated Samd2/3‐Nkx‐2.5 and ‐Ekr1/2 signalling activation in CFs during cardiac remodelling and fibrosis in post‐MI. On the contrary, increasing evidence indicated that HDAC6/ TGF‐β1‐TGF‐βR1 can modulate M2 macrophage polarization in cardiovascular tissues in various pathological conditions.^35^, ^36^ Our observation here show that HDAC6 inhibition lowered the CD206 (M2 macrophage marker) protein expression in the infracted myocardium, indicating that the impaired M2 polarization might be due to the reduction of HDAC6/ TGF‐β1‐TGF‐βR1 in post‐MI heart.

Myocardial infarction‐related heart failure (HF) is characterized by a progressive loss of cardiomyocytes, manifested by ventricular chamber remodelling and the accumulation of interstitial fibrosis, leading to reduced cardiac output.^37^ Progressive cardiac fibrosis in heart failure is mediated primarily by tissue‐resident cardiac fibroblasts that become activated and differentiated into a different cell type, that is, myofibroblasts.^38^, ^39^ Fibroblast activation in the heart is induced by inflammatory cytokines, neuroendocrine agonists, ventricular pressure overload or MI injury. Myofibroblasts produce and secrete abundant ECM substances and also acquire contractile activity through the induction of genes such as α‐SMA, allowing these cells to physically remodel the scar after MI.^40^, ^41^

TGF‐β is thought to activate fibroblasts and promote the production of ECM,^42^ which binds to receptors on the plasma membrane and induces the phosphorylation of the Smad2/3 transcription factor, thereby mediating TGF‐β1/Smad2/3 signalling.^43^ The activation of collagen synthesis and secretion then begins, which can lead to increasing scar formation.^44^ Our present results demonstrated that genetic and pharmacological interventions against HADC6 markedly lowered the expressions of TGF‐β1, α‐SMA, collagen I and collagen II genes and proteins in the LV myocardium of post‐MI mice. HDAC6 has been shown to upregulate the TGF‐β1‐Smad2/3 and ‐Smad7 signalling pathways, leading to renal and pulmonary fibrosis.^45^, ^46^ Taken together, these data provide evidence that in mice under our experimental conditions, the cardiac protective actions of HDAC6 inhibition are mediated, at least in part, through TGF‐β1/Smad2/3‐dependent collagen synthesis. It should be noted that in lacking HDAC6 high myofibril stiffness can occur, leading to cardiac myofibrillar protein (composed of sarcomeric protein) acetylation and altered worsened diastolic dysfunction^47^ in the UNX/DOCA model. Oppositely, in 2024, Ranjbarvaziri and colleagues reported that HDAC6 lacking mice protected heart against stress of high fat‐diet and nitric oxide inhibition, and established diastolic dysfunction in HDAC6^+/+^ mice was rescued by HDAC6 inhibition with TYA‐018.^48^ The authors identified four lysine sites—K32104, K19868, K24707 and K31877—in titin that exhibited increased acetylation levels in diastolic HF mice treated with TYA‐018 compared to diastolic HF animals receiving the vehicle treatment. Notably, among these four sites, K32104 acetylation demonstrated a reduction in diastolic HF mice, which was subsequently reversed and increased following TYA‐018 treatment. The differential acetylation patterns observed in titin might, in part, elucidate the contrasting outcomes observed in different model systems of both studies. Based on the clinical/experimental context, deleting and inhibiting HDAC6 may result in both beneficial and detrimental effects in diastolic HF. It will be needed to set laboratory and clinical studies for fully exploring this issue.

Study limitations should be considered. First, we were unable to create cardiac cell (i.e. cardiac fibroblasts and cardiomyocytes) specific HADC6 knockout mice to fully clarify its role(s) in cardiac fibrosis and dysfunction to MI injury. And we also did not examine its functions in cardiac vascular smooth muscle proliferation and macrophage polarization in post‐MI heart. Second, we could not provide the direct evidence of the cellular crosstalk of HDAC6 in post‐MI heart. Third, unfortunately, we have not designed to insightfully compare the differences in the myofibril stiffness, diastolic dysfunction, cardiac rupture and prognosis between HDAC6^+/+^‐MI, HDAC6^−/−^‐MI and HDAC6^+/+^‐TubA MI mice. And there is no data of baseline echocardiography at day 1 after MI surgery. Further research is necessary to investigate these issues.

## 5 CONCLUSIONS

In summary, our present findings demonstrated that HADC6 deficiency resists ischemic injury by a reduction of TGF‐β1/Smad2/3 signalling activation, leading to decreased extracellular matrix production, which reduces cardiac fibrosis and dysfunction, providing a potential molecular target in the treatment of patients with MI.

## AUTHOR CONTRIBUTIONS

**Junqiao Fang:** Conceptualization (lead); data curation (lead); formal analysis (equal); investigation (lead); methodology (lead); project administration (lead); writing – original draft (lead). **Shangzhi Shu:** Resources (supporting); supervision (supporting); validation (supporting); visualization (supporting). **Hui Dong:** Methodology (supporting); resources (supporting); visualization (supporting). **Xueling Yue:** Resources (supporting); software (supporting); visualization (supporting). **Jinshun Piao:** Methodology (supporting); visualization (supporting). **Shuyan Li**: writing–review and editing (Supporting). **Lan Hong:** Resources (supporting). **Xian Wu Cheng:** Conceptualization (equal); data curation (equal); funding acquisition (equal); supervision (lead).

## FUNDING INFORMATION

This work was partly supported by grants from the National Natural Science Foundation of China (nos. 81770485 and 82370424 to XWC; no. 82350065 to LH; no. 82070299).

## CONFLINCT OF INTEREST STATEMENT

No conflicts of interest, financial or otherwise, are declared by the authors.

## Data Availability

The data underlying this article will be shared on reasonable request to the corresponding author.

## References

[1] GBD 2019 Diseases and Injuries Collaborators . Global burden of 369 diseases and injuries in 204 countries and territories, 1990‐2019: a systematic analysis for the global burden of disease study 2019. Lancet. 2020;396:1204‐1222. doi:10.1016/s0140-6736(20)30925-9 33069326 PMC7567026

[2] Mensah GA , Roth GA , Fuster V . The global burden of cardiovascular diseases and risk factors: 2020 and beyond. J Am Coll Cardiol. 2019;74:2529‐2532. doi:10.1016/j.jacc.2019.10.009 31727292

[3] Roth GA , Mensah GA , Johnson CO , et al. Global burden of cardiovascular diseases and risk factors, 1990–2019: update from the GBD 2019 study. J Am Coll Cardiol. 2020;76:2982‐3021. doi:10.1016/j.jacc.2020.11.010 33309175 PMC7755038

[4] Talman V , Ruskoaho H . Cardiac fibrosis in myocardial infarction‐from repair and remodeling to regeneration. Cell Tissue Res. 2016;365:563‐581. doi:10.1007/s00441-016-2431-9 27324127 PMC5010608

[5] Nahrendorf M , Pittet MJ , Swirski FK . Monocytes: protagonists of infarct inflammation and repair after myocardial infarction. Circulation. 2010;121:2437‐2445. doi:10.1161/circulationaha.109.916346 20530020 PMC2892474

[6] Frangogiannis NG . Regulation of the inflammatory response in cardiac repair. Circ Res. 2012;110:159‐173. doi:10.1161/circresaha.111.243162 22223212 PMC3690135

[7] Peserico A , Simone C . Physical and functional HAT/HDAC interplay regulates protein acetylation balance. J Biomed Biotechnol. 2011;2011:371832. doi:10.1155/2011/371832 21151613 PMC2997516

[8] Haberland M , Montgomery RL , Olson EN . The many roles of histone deacetylases in development and physiology: implications for disease and therapy. Nat Rev Genet. 2009;10:32‐42. doi:10.1038/nrg2485 19065135 PMC3215088

[9] Kumar S . Drug targets for cancer treatment: an overview. Med Chem. 2015;5:115. doi:10.4172/2161-0444.1000252

[10] Gregoretti IV , Lee YM , Goodson HV . Molecular evolution of the histone deacetylase family: functional implications of phylogenetic analysis. J Mol Biol. 2004;338:17‐31. doi:10.1016/j.jmb.2004.02.006 15050820

[11] Lee TM , Lin MS , Chang NC . Inhibition of histone deacetylase on ventricular remodeling in infarcted rats. Am J Physiol Heart Circ Physiol. 2007;293:H968‐H977. doi:10.1152/ajpheart.00891.2006 17400721

[12] Mani SK , Kern CB , Kimbrough D , et al. Inhibition of class I histone deacetylase activity represses matrix metalloproteinase‐2 and ‐9 expression and preserves LV function postmyocardial infarction. Am J Physiol Heart Circ Physiol. 2015;308:H1391‐H1401. doi:10.1152/ajpheart.00390.2014 25795711 PMC4451303

[13] Lemon DD , Horn TR , Cavasin MA , et al. Cardiac HDAC6 catalytic activity is induced in response to chronic hypertension. J Mol Cell Cardiol. 2011;51:41‐50. doi:10.1016/j.yjmcc.2011.04.005 21539845 PMC3113526

[14] Demos‐Davies KM , Ferguson BS , Cavasin MA , et al. HDAC6 contributes to pathological responses of heart and skeletal muscle to chronic angiotensin‐II signaling. Am J Physiol Heart Circ Physiol. 2014;307:H252‐H258. doi:10.1152/ajpheart.00149.2014 24858848 PMC4101640

[15] Györfi AH , Matei AE , Distler JHW . Targeting TGF‐β signaling for the treatment of fibrosis. Matrix Biol. 2018;68‐69:8‐27. doi:10.1016/j.matbio.2017.12.016 29355590

[16] Butler KV , Kalin J , Brochier C , Vistoli G , Langley B , Kozikowski AP . Rational design and simple chemistry yield a superior, neuroprotective HDAC6 inhibitor, tubastatin A. J Am Chem Soc. 2010;132:10842‐10846. doi:10.1021/ja102758v 20614936 PMC2916045

[17] Gao E , Lei YH , Shang X , et al. A novel and efficient model of coronary artery ligation and myocardial infarction in the mouse. Circ Res. 2010;107:1445‐1453. doi:10.1161/CIRCRESAHA.110.223925 20966393 PMC3005817

[18] Jin X , Yue X , Huang Z , et al. Cathepsin K deficiency prevented stress‐related thrombosis in a mouse FeCl(3) model. Cell Mol Life Sci. 2024;81:205. doi:10.1007/s00018-024-05240-0 38703204 PMC11069486

[19] Zhang M , Yue X , Xu S , et al. Dipeptidyl peptidase‐4 disturbs adipocyte differentiation via the negative regulation of the glucagon‐like peptide‐1/adiponectin‐cathepsin K axis in mice under chronic stress conditions. FASEB J. 2024;38:e23684. doi:10.1096/fj.202400158R 38795334

[20] Piao L , Huang Z , Inoue A , Kuzuya M , Cheng XW . Human umbilical cord‐derived mesenchymal stromal cells ameliorate aging‐associated skeletal muscle atrophy and dysfunction by modulating apoptosis and mitochondrial damage in SAMP10 mice. Stem Cell Res Ther. 2022;13:226. doi:10.1186/s13287-022-02895-z 35659361 PMC9166592

[21] Wan Y , Piao L , Xu S , et al. Cathepsin S activity controls chronic stress‐induced muscle atrophy and dysfunction in mice. Cell Mol Life Sci. 2023;80:254. doi:10.1007/s00018-023-04888-4 37589754 PMC10435624

[22] Benoy V , Van Helleputte L , Prior R , et al. HDAC6 is a therapeutic target in mutant GARS‐induced Charcot‐Marie‐tooth disease. Brain. 2018;141:673‐687. doi:10.1093/brain/awx375 29415205 PMC5837793

[23] Wu H , Cheng XW , Hu L , et al. Cathepsin S activity controls injury‐related vascular repair in mice via the TLR2‐mediated p38MAPK and PI3K‐Akt/p‐HDAC6 signaling pathway. Arterioscler Thromb Vasc Biol. 2016;36:1549‐1557. doi:10.1161/atvbaha.115.307110 27365406 PMC4961274

[24] Yang J , Grafton F , Ranjbarvaziri S , et al. Phenotypic screening with deep learning identifies HDAC6 inhibitors as cardioprotective in a BAG3 mouse model of dilated cardiomyopathy. Sci Transl Med. 2022;14:eabl5654. doi:10.1126/scitranslmed.abl5654 35857625

[25] Cheng XW , Narisawa M , Wang H , Piao L . Overview of multifunctional cysteinyl cathepsins in atherosclerosis‐based cardiovascular disease: from insights into molecular functions to clinical implications. Cell Biosci. 2023;13:91. doi:10.1186/s13578-023-01040-4 37202785 PMC10197855

[26] Wang H , Inoue A , Lei Y , Wu H , Hong L , Cheng XW . Cathepsins in the extracellular space: focusing on non‐lysosomal proteolytic functions with clinical implications. Cell Signal. 2023;103:110531. doi:10.1016/j.cellsig.2022.110531 36417977

[27] Chen H , Wang J , Xiang MX , et al. Cathepsin S‐mediated fibroblast trans‐differentiation contributes to left ventricular remodelling after myocardial infarction. Cardiovasc Res. 2013;100:84‐94. doi:10.1093/cvr/cvt158 23771947 PMC3778959

[28] Weng L , Ye J , Yang F , et al. TGF‐β1/SMAD3 regulates programmed cell death 5 that suppresses cardiac fibrosis post‐myocardial infarction by inhibiting HDAC3. Circ Res. 2023;133:237‐251. doi:10.1161/circresaha.123.322596 37345556

[29] Ragazzini S , Scocozza F , Bernava G , et al. Mechanosensor YAP cooperates with TGF‐β1 signaling to promote myofibroblast activation and matrix stiffening in a 3D model of human cardiac fibrosis. Acta Biomater. 2022;152:300‐312. doi:10.1016/j.actbio.2022.08.063 36055606

[30] Pinnamaneni JP , Singh VP , Kim MB , et al. p63 silencing induces epigenetic modulation to enhance human cardiac fibroblast to cardiomyocyte‐like differentiation. Sci Rep. 2022;12:11416. doi:10.1038/s41598-022-15559-y 35794145 PMC9259667

[31] Bachamanda Somesh D , Klose K , Maring JA , et al. Cardiomyocyte precursors generated by direct reprogramming and molecular beacon selection attenuate ventricular remodeling after experimental myocardial infarction. Stem Cell Res Ther. 2023;14:296. doi:10.1186/s13287-023-03519-w 37840130 PMC10577947

[32] Maioli M , Santaniello S , Montella A , et al. Hyaluronan esters drive Smad gene expression and signaling enhancing cardiogenesis in mouse embryonic and human mesenchymal stem cells. PLoS One. 2010;5:e15151. doi:10.1371/journal.pone.0015151 21152044 PMC2994904

[33] Ulm S , Liu W , Zi M , et al. Targeted deletion of ERK2 in cardiomyocytes attenuates hypertrophic response but provokes pathological stress induced cardiac dysfunction. J Mol Cell Cardiol. 2014;72:104‐116. doi:10.1016/j.yjmcc.2014.03.002 24631771 PMC4046245

[34] Zhang L , Deng M , Lu A , et al. Sodium butyrate attenuates angiotensin II‐induced cardiac hypertrophy by inhibiting COX2/PGE2 pathway via a HDAC5/HDAC6‐dependent mechanism. J Cell Mol Med. 2019;23:8139‐8150. doi:10.1111/jcmm.14684 31565858 PMC6850921

[35] Wu J , Jiang L , Wang S , Peng L , Zhang R , Liu Z . TGF β1 promotes the polarization of M2‐type macrophages and activates PI3K/mTOR signaling pathway by inhibiting ISG20 to sensitize ovarian cancer to cisplatin. Int Immunopharmacol. 2024;134:112235. doi:10.1016/j.intimp.2024.112235 38761779

[36] Xu G , Niu L , Wang Y , et al. HDAC6‐dependent deacetylation of TAK1 enhances sIL‐6R release to promote macrophage M2 polarization in colon cancer. Cell Death Dis. 2022;13:888. doi:10.1038/s41419-022-05335-1 36270986 PMC9587286

[37] Benjamin EJ , Blaha MJ , Chiuve SE , et al. Heart disease and stroke Statistics‐2017 update: a report from the American Heart Association. Circulation. 2017;135:e146‐e603. doi:10.1161/cir.0000000000000485 28122885 PMC5408160

[38] Souders CA , Bowers SL , Baudino TA . Cardiac fibroblast: the renaissance cell. Circ Res. 2009;105:1164‐1176. doi:10.1161/circresaha.109.209809 19959782 PMC3345531

[39] Takeda N , Manabe I , Uchino Y , et al. Cardiac fibroblasts are essential for the adaptive response of the murine heart to pressure overload. J Clin Invest. 2010;120:254‐265. doi:10.1172/jci40295 20038803 PMC2798693

[40] Hinz B . The myofibroblast: paradigm for a mechanically active cell. J Biomech. 2010;43:146‐155. doi:10.1016/j.jbiomech.2009.09.020 19800625

[41] Wynn TA . Cellular and molecular mechanisms of fibrosis. J Pathol. 2008;214:199‐210. doi:10.1002/path.2277 18161745 PMC2693329

[42] Meng XM , Nikolic‐Paterson DJ , Lan HY . TGF‐β: the master regulator of fibrosis. Nat Rev Nephrol. 2016;12:325‐338. doi:10.1038/nrneph.2016.48 27108839

[43] Derynck R , Zhang YE . Smad‐dependent and Smad‐independent pathways in TGF‐beta family signalling. Nature. 2003;425:577‐584. doi:10.1038/nature02006 14534577

[44] Bowers SL , Banerjee I , Baudino TA . The extracellular matrix: at the center of it all. J Mol Cell Cardiol. 2010;48:474‐482. doi:10.1016/j.yjmcc.2009.08.024 19729019 PMC2824065

[45] Chen X , Yu C , Hou X , et al. Histone deacetylase 6 inhibition mitigates renal fibrosis by suppressing TGF‐β and EGFR signaling pathways in obstructive nephropathy. Am J Physiol Renal Physiol. 2020;319:F1003‐F1014. doi:10.1152/ajprenal.00261.2020 33103445 PMC7792693

[46] Li Y , Qin W , Liang Q , et al. Bufei huoxue capsule alleviates bleomycin‐induced pulmonary fibrosis in mice via TGF‐β1/Smad2/3 signaling. J Ethnopharmacol. 2023;316:116733. doi:10.1016/j.jep.2023.116733 37277082

[47] Lin YH , Major JL , Liebner T , et al. HDAC6 modulates myofibril stiffness and diastolic function of the heart. J Clin Invest. 2022;132:e148333. doi:10.1172/jci148333 35575093 PMC9106344

[48] Ranjbarvaziri S , Zeng A , Wu I , et al. Targeting HDAC6 to treat heart failure with preserved ejection fraction in mice. Nat Commun. 2024;15:1352. doi:10.1038/s41467-024-45440-7 38409164 PMC10897156

